# Experimental and Numerical Analysis of a Reinforced Wood Lap Joint

**DOI:** 10.3390/ma13184117

**Published:** 2020-09-16

**Authors:** Lingpeng Ye, Baisheng Wang, Pujian Shao

**Affiliations:** 1 College of Civil Engineering and Architecture, Zhejiang University, Hangzhou 310058, China; ylpccea@zju.edu.cn; 2 Institute of Cultural Relics and Archaeology, Hangzhou 310014, China; xspj06@163.com

**Keywords:** ancient wood structures, wood, beam, lap joint, reinforcement, CFRP, bearing capacity, failure mode, numerical analysis

## Abstract

During the restoration of ancient wood structures, the original material of the structures should be kept as much as possible, so a spliced method by using lap joints is commonly used to repair ancient wood structures. This study studies the mechanical behavior of a lap joint which was reinforced with fiber composite materials or steels. An experimental and numerical analysis were performed to study the strength, stiffness and failure modes of the lap joints. The test results showed that the strengthening effect of sticking carbon fiber-reinforced polymer (CFRP) sheets is better than that of sticking CFRP bars or steel bars due to the better bonding conditions; therefore, the lap joint reinforced with CFRP sheets was further analyzed using a numerical approach. The strength and stiffness were enhanced by increasing the reinforcement ratio of CFRP sheets. The use of a 0.34% reinforcement ratio made the bearing capacity of the lap joint reach that of the intact beam. The numerical model agreed well with the experiments in terms of stiffness. By analyzing the numerical analysis results, the structural behavior of the lap joint was revealed. A numerical model can be used to predict the stiffness and behavior of spliced beams with lap joints of different sizes.

## 1. Introduction

There are many ancient wood structures in China, which present China’s material and spiritual civilization. However, due to the impact of the natural environment and human factors, the wood components of ancient wood structures are easily cracked, deformed, decayed and moth-eaten. In order to preserve most of original material, the lap joint is commonly used to replace the damaged part of a wood component. The core principle of architectural heritage protection is authenticity and integrity [1]. During the restoration of the ancient wood structures, the intervention on the original building should be minimized, the original material of the building should be kept as much as possible, and the value of cultural relics of the building should be protected as much as possible [2]. Partial component replacement with a lap joint is consistent with modern conservation principles of minimum intervention [3].

**Figure 1:**
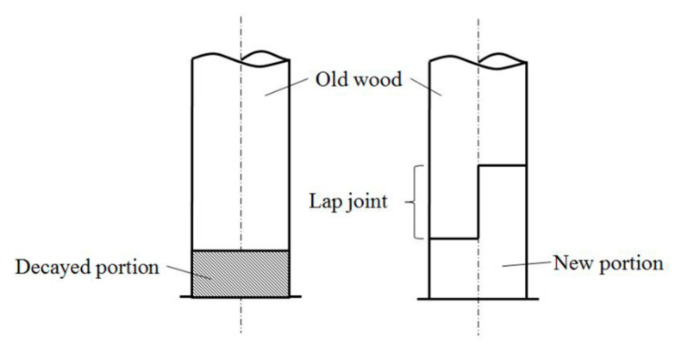
Schematic diagram of the spliced method for a timber column.

**Figure 2:**
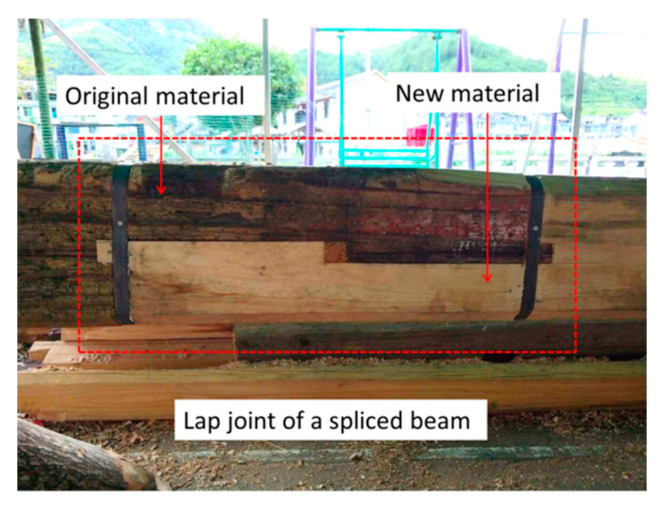
A lap joint of a spliced beam.

Arciszewska-Kedzior et al. and Hasníková et al. used a lapped scarf joint with inclined faces and wood dowels to repair the beam-end in historical timber beams, and investigated the mechanical behavior of the lapped scarf joint by an experimental and numerical model [4,5]. Li et al. used self-tapping wood screws to reinforce the lap joint in a spliced beam [6]. Sangree et al. examined the behavior of a halved and tabled traditional timber scarf joint [7,8]. Jensen et al. studied the shear strength of beam spliced joints with glued-in rods [9]. In China, when the bottom of a column in an ancient wood structure is seriously decayed, the decayed portion is replaced with a new portion (Figure 1), and the new portion is connected with the rest of the column by a lap joint [10]. This type of lap joint was also planned to be used for the restoration of the timber arch lounge bridges in China (Figure 2). The main bearing structure of the timber arch lounge bridge is a woven arch, which is composed of round wood components, and these round wood components need to bear loads such as the structure’s own weight and pedestrian loads [11,12]. However, the bearing capacity of an unreinforced lap joint could not reach the bearing capacity of the intact beam, which made it unsafe to be used in some load-bearing members.

Fiber-reinforced polymer (FRP) composite materials are well-known for possessing outstanding specific stiffness and strength, and FRP composite materials are widely used in the aviation industry, automobile industry and construction industry [13,14,15,16,17,18]. In terms of reinforcement and repair of wooden structures, steel materials and composite materials are widely used to reinforce the wood components. Many scholars used carbon fiber-reinforced polymer (CFRP) bars, CFRP sheets, steel bars or glass fiber-reinforced polymer (GFRP) to strengthen glulam beams [19,20,21,22,23]. Steel materials are increasingly being replaced by composite materials due to the advantages of light weight, high strength and corrosion resistance of composite materials [24,25,26]. Dewey et al. used CFRP strips and GFRP bars to strengthen the deteriorated timber bridge girders, and the results showed that the moment capacity and ductility of the repaired members were enhanced [27]. Svecova and Eden used GFRP bars to strengthen timber stringers. Strengthening timber stringers with GFRP reinforcement increased the ultimate strength of the stringers and reduced their variability [28]. Borri et al. performed a study on the reinforcement of existing wood elements under bending loads through the use of FRP materials [29]. Bergner et al. used carbon fiber and basalt fiber rovings to reinforce spruce wood specimens, and two methods (vacuum infusion process and TowPregs) were developed to manufacture the composites [30]. Qin performed an excellent study on using GFRP to strengthen spliced wood beams with lap joints, and studied the bending strength and shear strength of reinforced spliced beams by experiments [31]. Steel jackets and CFRPs were used to reinforce splice-jointed timber columns [32,33,34].

This paper studies the mechanical behavior of the half beam height lap joint reinforced with CFRP sheets, CFRP bars or steel bars. Two research methods, experimental and numerical analysis, were used in this paper. Intact beams and spliced beams with strengthening lap joints were tested to analyze the bearing capacity, stiffness and failure modes. The parameters that influence the mechanical behavior of the lap joint such as the reinforcement ratio and bond length of CFRP sheets will be discussed. The stress distribution and deformation of the lap joint reinforced by CFRP sheets will be studied by numerical analysis. Finally, a proposal for repairing wood beams with the lap joint is proposed.

## 2. Materials and Methods

This paper conducted experiments on spliced beams with different reinforcement methods. The lap joints of the spliced beams were reinforced with CFRP sheets, CFRP bars and steel bars. The lap joints reinforced with CFRP sheets considered different thicknesses (reinforcement ratio) and bonding lengths of CFRP sheets. To provide reference data for spliced beams with joints, two intact beams were also tested. Then this paper analyzed the spliced beams reinforced with CFRP sheets through numerical models. The numerical model was validated by experimental data. The numerical results were used to study the structural behavior of the lap joint.

### 2.1. Test Beams

The test beams in this paper were round beams. The beams were divided into two groups according to whether they mainly bear bending moments or shear forces. According to the actual conditions, seven bending beams (B0 to B6) and three shearing beams (S0 to S2) were prepared and tested. Beam B0 and beam S0 were intact beams. Beams B1 to B6 and beams S1 to S2 were spliced beams.

The main parameters of 10 beams are listed in Table 1. The diameter values in Table 1 refer to the dimensional values at the lap joints. The lap joints of the bending beams were set at the midspan, and the lap joints of shearing beams were set at one end of the beams. A pair of through-bolts with a diameter of 12 mm was set at the centerline of every lap joint. Two through-bolts were 150 mm apart and were symmetrically arranged.

**Figure 3:**
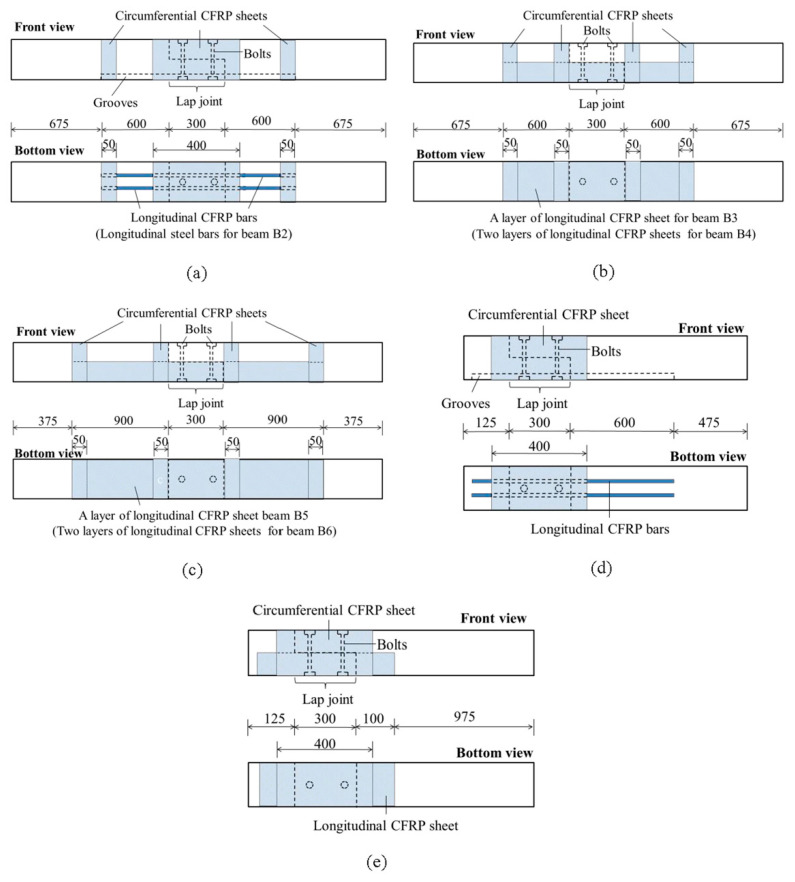
Schematic diagrams of the strengthening methods (in millimeters). (**a**) Beams B1 and B2; (**b**) beams B3 and B4; (**c**) beams B5 and B6; (**d**) beam S1; (**e**) beam S2.

Figure 3a shows the design drawing of beams B1 and B2. Beam B1 was reinforced using near surface mounted CFRP bars. Two grooves that were 12 mm deep, 12 mm wide and 1500 mm long were cut out along the bottom surface symmetrically. Two CFRP bars with a cross-sectional size of 10 mm × 10 mm were stuck to the grooves through a bonding agent. The CFRP bars extended 300 mm outside the region of pure bending. A layer of circumferential CFRP sheet (400 mm wide × 0.167 mm thick) was wrapped around the spliced joints, and a layer of circumferential CFRP sheet (50 mm wide × 0.167 mm thick) was wrapped around the sections near the ends of the CFRP bars. The only difference between beam B1 and beam B2 was that beam B2 was reinforced using steel square bars.

Figure 3b,c show the design drawings of beams B3 to B6. Beam B3 was reinforced by sticking a layer of 200 mm wide, 0.167 mm thick and 1500 mm long longitudinal CFRP sheet. The CFRP sheet was stuck on the tension face of the beam with a bonding agent. The bonding surface of the beam needed to be smoothed with sandpaper before sticking the CFRP sheet on the beam. A layer of 50 mm wide and 0.167 mm thick circumferential CFRP sheet was wrapped around the ends of the lap joint and the ends of longitudinal CFRP sheet, respectively. The longitudinal CFRP sheet of beam B3 extended 300 mm outside the region of pure bending. The difference between beam B3 and beam B5 was the bonding length. The longitudinal CFRP sheet of beam B5 extended 600 mm outside the region of pure bending. Beams B4 and B6 were reinforced with two layers of 200 mm wide and 0.167 mm thick longitudinal CFRP sheets. Except the thickness of the CFRP sheet, beam B4 was the same as beam B3, and beam B6 was the same as beam B5.

Figure 3d,e show the design drawings of beam S1 and beam S2. The lap joint of the shearing beam was set at one end of the beam. A layer of 400 mm wide and 0.167 mm thick circumferential CFRP sheet was wrapped around the lap joints of beam S1 and beam S2, respectively, to bear the shear force. The lap joints of beam S1 and beam S2 also need to bear small bending moments. Therefore, two CFRP bars were set into square bottomed 12 mm × 12 mm (±3 mm) channels on the tension face of beam S1 to bear the bending moment. Different from beam S1, a layer of 200 mm wide and 0.167 mm thick longitudinal CFRP sheet was stuck on the bottom of B2 to bear the bending moment.

### 2.2. Material Properties

**Figure 4:**
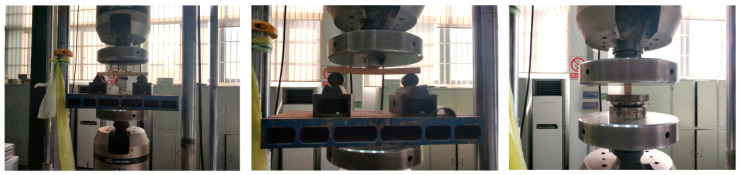
Material properties tests of Chinese fir.

The wood of the test beams was Chinese fir (Cunninghamia lanceolata (Lamb.) Hook). The Chinese fir used for tests was carried from south of Zhejiang Province, approximately 450 km south of Hangzhou. Wood is an anisotropic material. In order to study the material parameters of the wood used in this article, some material experiments were performed in this study to obtain the flexural modulus of elasticity, bending strength, compressive strength parallel to grain and moisture content. The elastic modulus and bending strength were obtained by performing 4-point static bending load tests according to Chinese standards GB/T 1936.1 [35] and GB/T 1936.2 [36]. The axial compressive strength was obtained by axial compression tests according to Chinese standard GB/T 1935 [37]. The moisture content was tested according to Chinese standard GB/T 1931 [38]. Figure 4 shows the material properties tests of the Chinese fir and Table 2 lists the average material properties and standard deviation of the test results. 

The properties of CFRP, steel bars and the bonding agent were provided by the manufacturers. The properties of CFRP and steel bars are listed in Table 3 and Table 4. The bonding agent used in these tests was a two-component epoxy resin structural adhesive, and the material properties of the bonding agent are listed in Table 5.

### 2.3. Instrumentation and Test Procedure

**Figure 5:**
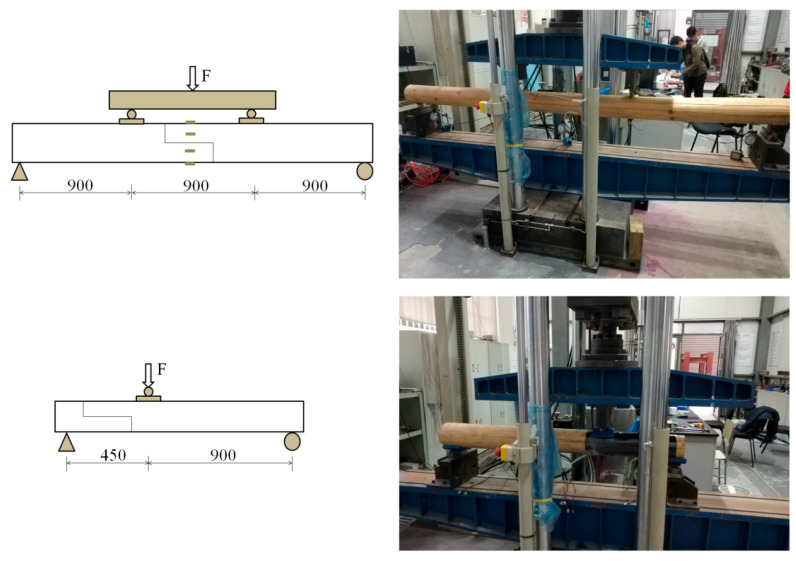
Loading device of load tests (in millimeters).

The bending tests were conducted using a four-point bending setup and the shear tests were conducted using a three-point bending setup (Figure 5). The tests were conducted on a testing machine with a capacity of 1000 kN. The make of the testing machine was INSTRON8805 (Instron Corporation, Canton, MA, USA). Vertical deflection at the lap joint was measured using linear variable displacement transducers (LVDTs, GRANDTEST, Guangzhou, China). For bending beams, six strain gauges with a gauge length of 30 mm were attached along the height of the section at the lap joints. All test data were recorded using a synchronous data acquisition system, and the frequency of data collection was 10 times per second.

### 2.4. Numerical Analysis

**Figure 6:**
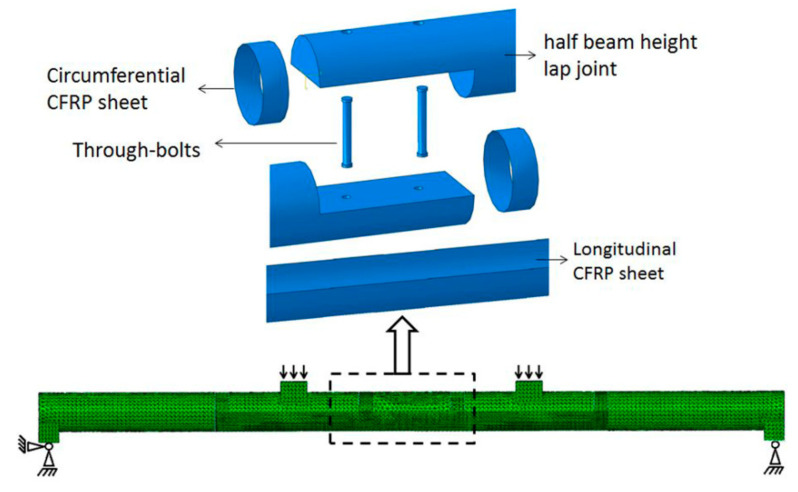
Numerical model of spliced beam with lap joint reinforced by CFRP sheets.

The numerical analysis aimed to investigate the structural behavior of the lap joint reinforced with CFRP sheets. In order to validate the numerical model of the lap joint reinforced with CFRP sheets, the numerical analysis results were compared with the experiment results. The numerical model was created in ABAQUS 6.11 (version 6.11, Dassault SIMULIA, Providence, RI, USA), and it was shown in Figure 6. The element type of wood and steel was C3D10, and the element type of CFRP sheet was S3. The load was applied symmetrically at the one-third span of the beam, and the load acted on the pad in the form of a uniform load. The constraints of the beam ends were hinged supports. The upper part of the lap joint and the lower part of the lap joint were contacted by hard contact in ABAQUS. The through-bolts and the lap joints were contacted by hard contact in ABAQUS. Bonding between the wood and CFRP sheets was very complicated and difficult to simulate, so CFRP sheets and wood were contacted by the tie constraint in ABAQUS. The material properties of steel and CFRP used the data in Table 3 and Table 4. Wooden material was modeled as linear elastic orthotropic material. As there was no condition to perform material experiments, the mechanical parameters of wooden materials used in the numerical model referred to existing research [39,40]. The mechanical parameters are shown in Table 6. The boundary condition of the beam ends was simply supported, which was consistent with the actual situation. According to the results of the mesh convergence analysis, the mesh size used in this study was 15 mm.

## 3. Results

### 3.1. Experimental Results of Bending Beams

**Figure 7:**
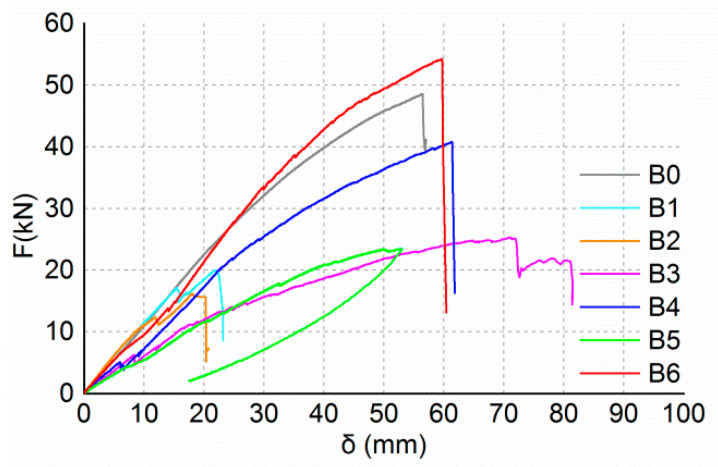
Load–deflection curves of bending beams.

**Figure 8:**
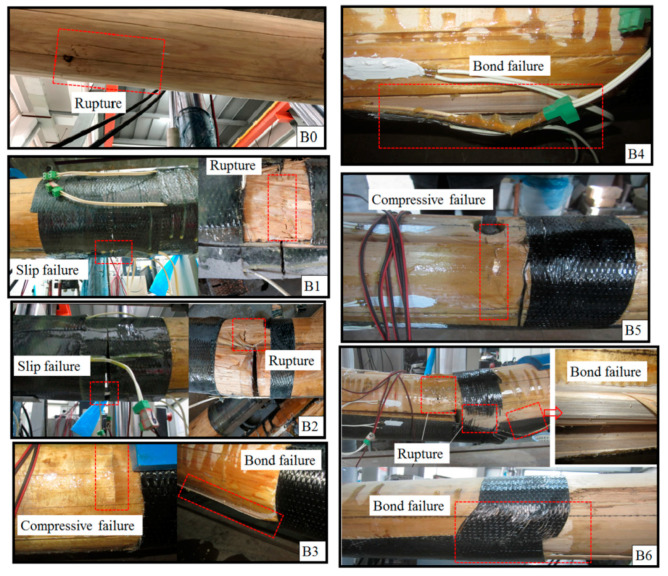
Failure modes of bending beams.

Table 7 shows the ultimate load values and the corresponding deflection of bending beams. Figure 7 shows the load–deflection curves of the bending beams. The failure modes of bending beams are shown in Figure 8.

The ultimate load value of beam B0 was 48.5 kN, and the corresponding deflection was 56.4 mm. When loaded to the ultimate load, beam B0 suddenly made a loud noise, and the timber on the tension side in the region of pure bending was split and ruptured (Figure 8). Before the beam ruptured, the load–deflection curve and strain–load curves were approximately linear.

When the load reached about 17.0 kN, slip failure of beam B1 occurred between the CFRP bars and the wood (Figure 8). When the slip failure happened, the deflection of the beam increased suddenly, and the load and strain decreased. The reason for this is that the surface of CFRP bars used in the tests was smooth and the bonding force between the bars and the wood was poor. Then, due to the strengthening effect of the through-bolts at the central axis of the lap joint, the load continued to increase. Finally, the upper piece of the lap joint was ruptured (Figure 8).

The performance of beam B2 was similar to that of beam B1. Slip failure between the steel bars and the wood occurred at a load of approximately 12.1 kN. The ultimate failure occurred due to the rupture of the upper piece of the lap joint (Figure 8).

The failure of beam B3 was different from beams B1 and B2. The compressive failure (Figure 8) of the timber on the top surface occurred firstly at a load of approximately 11.0 kN. Then, the stiffness of the beam decreased obviously and the strain of the longitudinal CFRP sheet increased rapidly. Ultimate failure of the beam occurred due to the debonding of the longitudinal CFRP sheet. Beam B3 had a large deflection of 70.8 mm, and the failure mode was ductile failure.

There was no obvious damage during the loading process of beam B4. The stiffness of the beam decreased at a load of approximately 25 kN, and tension strain of the longitudinal CFRP increased significantly. Ultimate failure occurred due to the bond failure between the longitudinal CFRP sheet and the wood (Figure 8).

The ultimate failure of beam B5 occurred due to the compressive failure of the timber on the top surface (Figure 8). The stiffness of the beam decreased at a load of approximately 19 kN, and strain of the longitudinal CFRP increased significantly. The load increased very slowly when it reached 23.4 kN, while the deflection increased rapidly.

The ultimate load value of beam B6 was larger than the ultimate load value of beam B0. Beam B6 failed due to the bond failure between the longitudinal CFRP sheet and the wood (Figure 8). Due to the large load, the lap joints were severely damaged, and both the circumferential CFRP sheet and the longitudinal CFRP sheet were ruptured.

**Figure 9:**
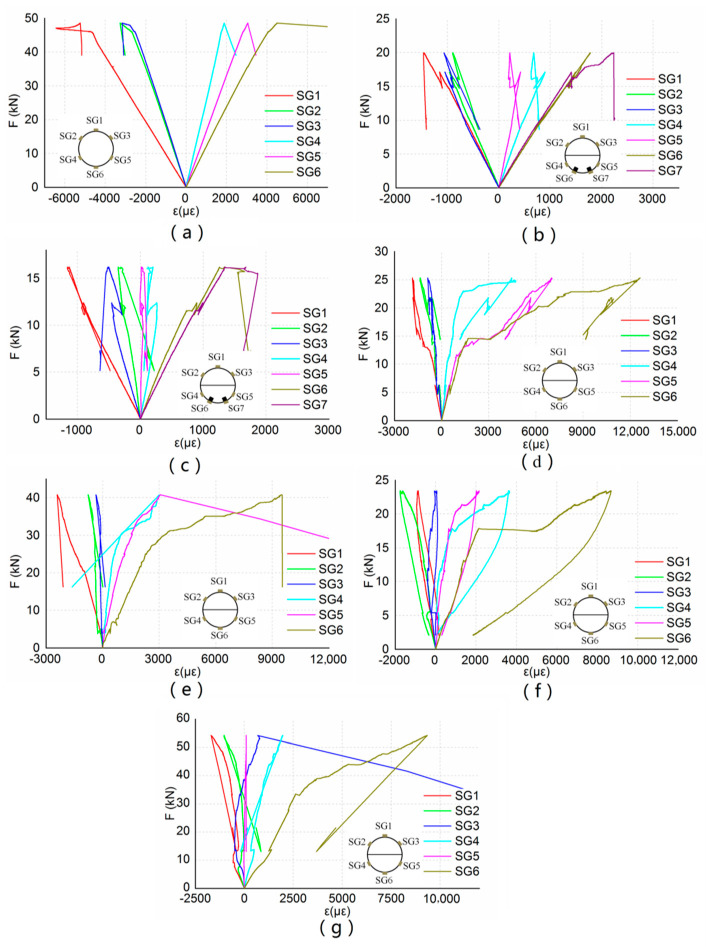
Load-strain diagram for bending beams. (**a**) Beam B0; (**b**) beam B1; (**c**) beam B2; (**d**) beam B3; (**e**) beam B4; (**f**) beam B5; (**g**) beam B6.

Figure 9 shows the load-strain diagrams of beams B0 to B6. The strains of CFRP bars and steel bars were small due to the small deflection. The maximum strain recorded at the ultimate load was 2254 and 1649 for beam B1 and beam B2, respectively. The strains of longitudinal CFRP sheets were larger than the strains of CFRP bars and steel bars due to the greater deflection of beams reinforced with CFRP sheets. For beam B3, the maximum strain of the longitudinal CFRP sheet recorded at the ultimate load was 12,250. When the stiffness of the beam decreased, the strain of the longitudinal CFRP sheet increased more quickly. The strain gauges were attached to the midspan sections, but the maximum compression strains appeared on the sections where the bolt holes were located. So, the non-linear strain behavior in the compression zone could not be recorded.

### 3.2. Experimental Results of Shearing Beams

**Figure 10:**
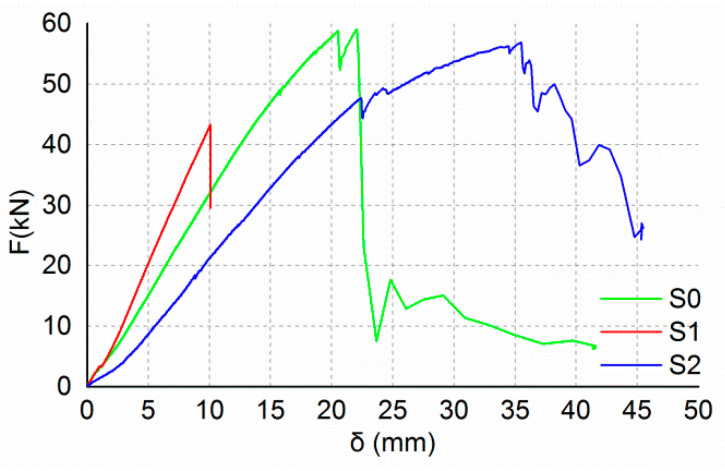
Load–deflection curves of shearing beams.

**Figure 11:**
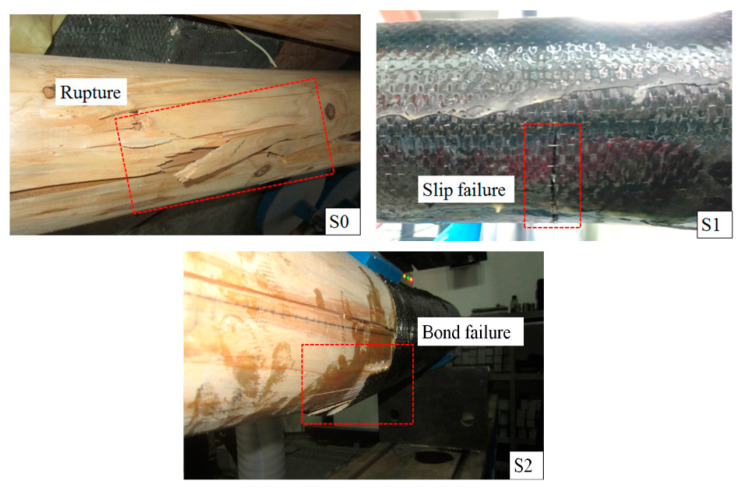
Failure modes of shearing beams.

Figure 10 shows the load–deflection curves of the shearing beams and Figure 11 shows the failure modes of the shearing beams. The test results showed that the failure modes of the shearing beams are bending failure. When the load reached 57.4 kN, beam S0 ruptured at the bottom of the cross-section at the loading position. The failure of beam S1 occurred due to the bond failure between the longitudinal CFRP bars and the wood. Similarly, the failure of beam S1 occurred due to the bond failure between the longitudinal CFRP sheet and the wood. The ultimate load values of beams S1 and S2 were 43.2 and 56.8 kN, respectively, which were approximately 75 percent and 99 percent of that of beam S0. The circumferential CFRP sheets of beams S1 and S2 were in good condition. The bond strength between the longitudinal CFRP sheet and wood determined the bearing capacity of the shearing beams.

### 3.3. Numerical Models

**Figure 12:**
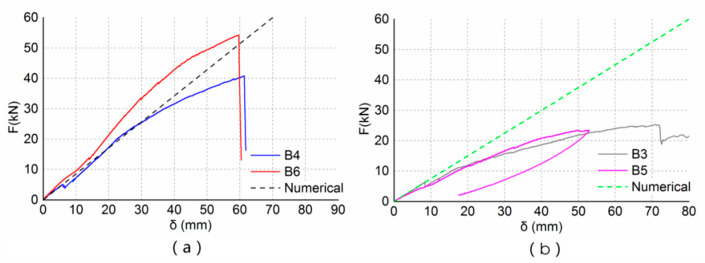
Comparison of experimental and numerical results. (**a**) Reinforced with two layers of CFRP sheets; (**b**) reinforced with one layer of CFRP sheet.

Figure 12 shows the load–deflection curves of experimental results and numerical results. As shown in Figure 12, the stiffness of the numerical model is close to the experimental results in the elastic stage. When reinforced with two layers of CFRP sheets, the stiffness of beam B4 was almost the same as the numerical model, and the stiffness of the numerical model was about 20% lower than that of beam B6. When reinforced with one layer of a CFRP sheet, the stiffness of the numerical model was about 18% greater than that of the experimental beams. The differences between the numerical and experimental results were caused by the deviations between the idealized model and the specimens. The wood material properties of the idealized model might have deviations from that of the specimens. There were inevitably construction errors in the process of specimen production, which led to deviations between the specimens and the idealized model. The idealized model could not simulate the bond slip relationship between CFRP and wood, which also could lead to deviations between the specimens and the idealized model. In general, the numerical model was in good agreement with the test results.

**Figure 13:**
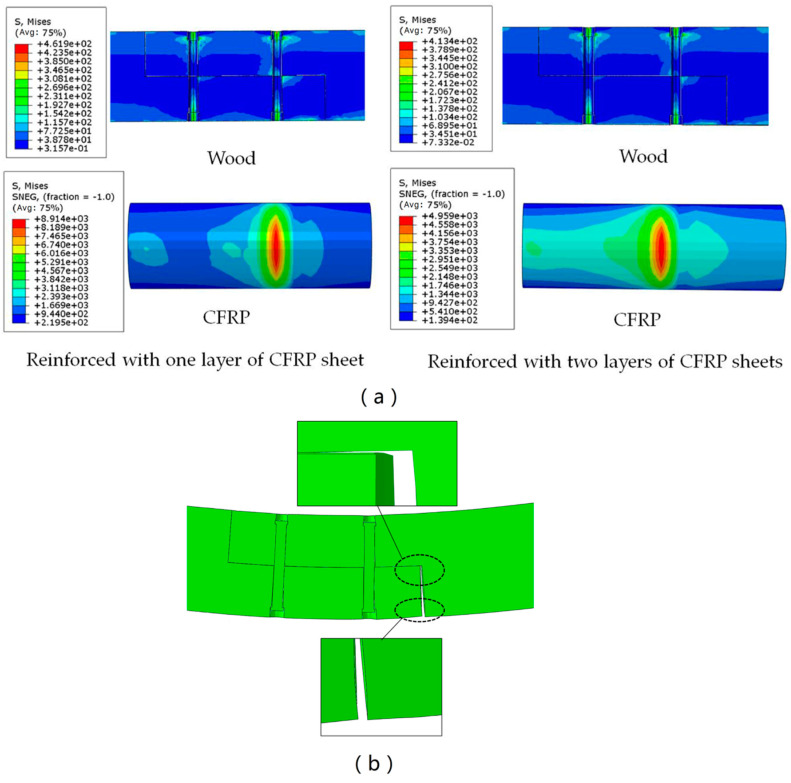
Stress contour and deformation of the lap joint. (**a**) Stress contour; (**b**) deformation.

The stress contour of the lap joints reinforced with CFRP sheets (Figure 13a) was shown to illustrate the stress distribution of the lap joint. As seen in Figure 13a, longitudinal CFRP sheets resisted the tension on the bottom of the lap joint. The maximum tension stresses occurred on the longitudinal CFRP sheet near the seam of the lap joint. The upper part of the lap joint resisted the compression on the top of the lap joint. The maximum compression stresses occurred near the bolt hole. Figure 13b shows the deformation of the lap joint. As seen in Figure 13b, the upper part and lower part of the lap joint were separated at the bottom seam of the lap joint. The seam deformation of the lap joint reinforced with one-layer of a CFRP sheet was about 1.35 mm, and the seam deformation of the lap joint reinforced with two layers of CFRP sheets was about 0.80 mm.

## 4. Discussion

Reinforced spliced beams with lap joints were tested in this paper. The test results show that the effect of reinforcement with CFRP sheets is better than the effect of reinforcement with CFRP bars or steel bars. The reason is that the bond strength between the CFRP sheets and wood is better than the bond strength between the CFRP bars and wood. The ultimate load value of beam B6 was about 111.5 percent of the ultimate load value of beam B0, and the ultimate load value of beam S2 was about 99 percent of the ultimate load value of beam S0. This shows that by affixing a sufficient thickness of longitudinal CFRP sheet at the bottom of the lap joint and providing reliable adhesion between the longitudinal CFRP sheet and the wood, the flexural load-bearing capacity of the beam with a lap joint can exceed the load-bearing capacity of the intact beam.

This paper used the finite element method to simulate the spliced beam reinforced by CFRP sheets, and a correlation between numerical results and experimental results is herein discussed. As shown in Figure 12, the differences between the numerical and experimental results are small. The differences in the stiffness between numerical and experimental values are of 20% and 18% for the reinforced beams with two layers of CFRP sheets and one layer of CFRP sheets, respectively. Taking into account the inevitable deviations between the numerical model and the specimens, the relative error of finite elemental models is low. The numerical model can be used to predict the stiffness of the specimens in the elastic stage. In addition, the finite elemental model can be used to analyze the structural behavior of the reinforced lap joints and explain some experimental results.

**Figure 14:**
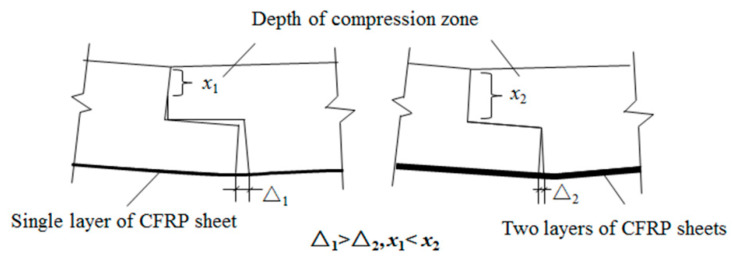
The deformation of the lap joint.

**Figure 15:**
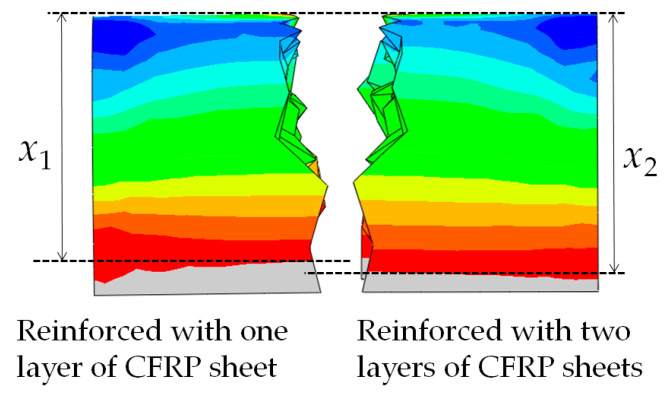
Comparison of the depth of compression zone.

**Figure 16:**
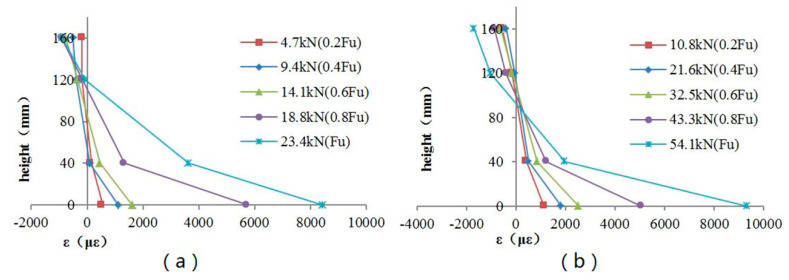
Strain profile for beams B5 and B6. (**a**) Beam B5 (reinforced with one-layer CFRP sheet); (**b**) beam B6 (reinforced with two layers of CFRP sheets).

The ultimate load value of beam B4 increased by about 61.5 percent compared to that of beam B3, and the ultimate load value of beam B6 increased by about 131.2 percent compared to that of beam B5. Comparison between the ultimate load values of beam B3 and beam B4 or beam B5 and beam B6 showed that increasing the thickness of the longitudinal CFRP sheet could significantly increase the bearing capacity of the lap joints. As shown in Figure 7, the stiffness of beams B4 and B6 are greater than the stiffness of beams B3 and B5. The stiffness of the beam also increased as the thickness of the longitudinal CFRP sheet increased. The reason why the stiffness and bearing capacity of the specimens reinforced with two layers of CFRP sheets are increased is that increasing the thickness of the CFRP sheet reduces the deformation of the lap joint and improves its mechanical properties. As seen in Figure 14, the seam in the lower half of the lap joint will separate. The numerical results showed that the seam deformation of the lap joint reinforced with one layer of a CFRP sheet (Δ1) was larger than that of the lap joint reinforced with two layers of CFRP sheets (Δ2). The seam deformation would weaken the integrity of the lap joint, which was not conducive to the bearing capacity of the joint. On the one hand, the depth of the compression zone of the lap joint will decrease as the thickness of the CFRP sheet decreases. As seen in Figure 15 and Figure 16, both numerical results and experimental results show that the depth of the compression zone of the specimen reinforced with one layer of CFRP sheet is smaller than that of the specimen reinforced with two layers of CFRP sheets. On the other hand, under the same load, the stress and strain of the specimens will increase as the thickness of the CFRP sheet decreases. As seen in Figure 16, in the case of similar loads, the maximum tensile strain and maximum compressive strain of beam B5 are both larger than the beam B6. The numerical results also show the same pattern (Figure 13).

The numerical results can also be used to explain why beams B3 and B5 failed due to the compressive failure of the wood on the top surface while beams B4 and B6 failed due to the bond failure between the CFRP sheet and the wood. As discussed above, the specimens reinforced with one layer of a CFRP sheet have greater stress and smaller depth of the compression zone. The smaller depth of the compression zone and greater stress make the upper section prone to compressive failure, which can explain why beams B3 and B5 failed due to the compressive failure. When reinforced with two layers of CFRP sheets, the depth of the compression zone of wood increases and the cross-sectional area involved in compression also increases, which can provide a resistance greater than the bonding force and make bonding failure occur before compression failure.

**Figure 17:**
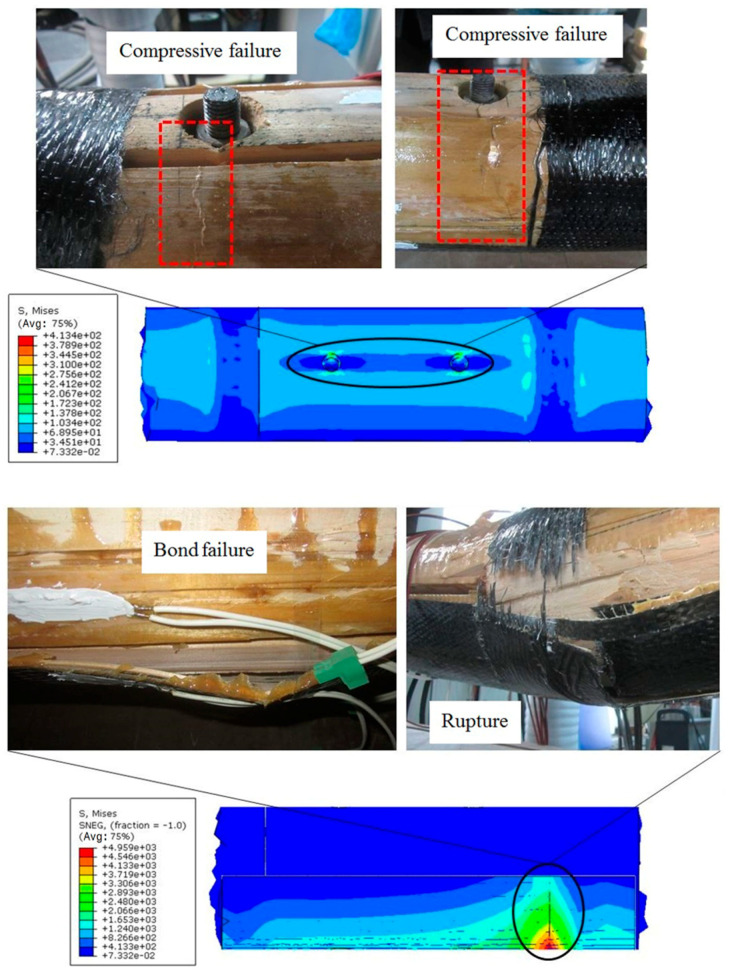
Comparison between the stress contour and the failure modes of the specimens.

In addition, the location of the maximum stress in the numerical models and the location of the failure in experiments are highly consistent, which reveals that the numerical models also can be used to predict possible failure modes. Figure 17 shows the comparison between the stress contour and the failure modes of the specimens. According to the stress contour, the maximum compression stress occurred around the bolt hole. Beams B3 and B5 failed due to the compressive failure of wood near the bolt hole where the compressive stress is the largest. The maximum tension stress occurred on the longitudinal CFRP sheet near the seam of the lap joint, so the bond failure between the CFRP sheet and wood happened first near the seam of the lap joint. The rupture of longitudinal CFRP sheet also occurred near the seam of the lap joint where the tension stress is the largest. Although the finite elemental model in this study cannot simulate the failure mode, it can better predict the location and form of the failure.

## 5. Conclusions

A CFRP sheet was used to strength the lap joints of the spliced beams in this article. The experimental results of the spliced beams with lap joints were discussed. The final conclusions reached were as follows:

1. The reinforcement effects of CFRP sheets were better than that of CFRP bars and steel bars. The ultimate loads of the lap joint reinforced with CFRP bars and steel bars were small due to the poor bond strength between the bars and wood. The failure modes of beams reinforced with CFRP bars or steel bars were slip failure between the bars and wood. The failure modes of beams reinforced with CFRP sheets were compressive failure or bond failure.
2. The numerical model in this paper could better simulate the mechanical behavior of the lap joint in this paper. It can be used to predict the mechanical properties of the lap joint with different dimensions and materials.
3. Increasing the reinforcement ratio could significantly increase the load-bearing capacity of the lap joint. The bending capacity of the lap joint with a low reinforcement ratio (0.34%) could reach the bending capacity of the intact beam. Moreover, using a higher reinforcement ratio could increase the integrality of the lap joint, and made the depth of the compression zone greater.
4. Increasing the bond strength between the longitudinal CFRP sheet and wood can increase the load-bearing capacity of the lap joint. If the bonding strength was sufficient, the lap joint will fail due to the compression failure. Otherwise, the lap joint will fail due to the bond failure.
5. The bending moment at the end of the wood beam is generally small, and the requirements for the bonding length and reinforcement ratio of the CFRP sheet on the tension face will be smaller, so it is recommended to set the lap joint at the end of the wood beam.

## Figures and Tables

**Table 1:** Main parameters of test beams.

Group	Label	Length of the Beam (mm)	Diameter (mm)	Length of Lap Joint (mm)	Reinforced Method	Reinforcement Ratio (%)
Bending beams	B0	2850	160	300	-	-
	B1	2850	168	300	CFRP bar	1.0
	B2	2850	167	300	steel bar	1.0
	B3	2850	167	300	CFRP sheet	0.17
	B4	2850	160	300	CFRP sheet	0.34
	B5	2850	158	300	CFRP sheet	0.17
	B6	2850	164	300	CFRP sheet	0.34
Shearing beams	S0	1510	168	300	-	-
	S1	1510	170	300	CFRP bar	1.0
	S2	1510	170	300	CFRP sheet	0.17

**Table 2:** Average material properties of the wood from own measurements.

Parameter	Average Value	Standard Deviation
Flexural modulus of elasticity (N/mm^2^)	8888.00	978.3
Bending strength (N/mm^2^)	68.44	5.7
Compressive strength parallel to grain (N/mm^2^)	36.40	1.8
Density (g/cm^3^)	0.45	-
Moisture content (%)	11.12	0.1

**Table 3:** Material properties of carbon fiber-reinforced polymer (CFRP) from the manufacturers.

Parameter	CFRP
Modulus of elasticity (10^3^ N/mm^2^)	230
Tensile strength (N/mm^2^)	3400
Bending strength (N/mm^2^)	700
Density (g/cm^3^)	1.77
Elongation at rupture (‰)	17.2

**Table 4:** Material Properties of steel bars from the manufacturers.

Parameter	Steel Bars
Modulus of elasticity (10^3^ N/mm^2^)	210
Tensile strength (N/mm^2^)	400
Yield Strength (N/mm^2^)	235
Density (g/cm^3^)	7.85
Elongation at rupture (%)	21.2

**Table 5:** Material properties of bonding agent from the manufacturers.

Parameter	Epoxy Resin
Modulus of elasticity in tension (N/mm^2^)	2400
Tensile strength (N/mm^2^)	59
Compressive strength (N/mm^2^)	80
Bending strength (N/mm^2^)	62
Elongation at break (%)	1.6
Shear strength (steel to steel) (N/mm^2^)	27

**Table 6:** Material properties for spliced beams in numerical model [39,40].

Species	Modulus of Elasticity (N/mm^2^)			Poisson’s Ratio			Shear Modulus(N/mm^2^)		
	E_L_	E_R_	E_T_	μ_LR_	μ_RT_	μ_LT_	G_LR_	G_RT_	G_LT_
Chinese fir	12,520	1018	572	0.1	0.35	0.1	1231	234	617

**Table 7:** The ultimate load values and the corresponding deflection of bending beams.

Label	Ultimate Load F_u_ (kN)	Deflection δ (mm)
B0	48.5	56.4
B1	20.0	21.9
B2	16.1	18.1
B3	25.2	70.8
B4	40.7	61.3
B5	23.4	53.0
B6	54.1	59.6
